# Simian Varicella Virus Pathogenesis in Skin during Varicella and Zoster

**DOI:** 10.3390/v14061167

**Published:** 2022-05-27

**Authors:** Ravi Mahalingam, Brittany Feia, Colin Coleman, Kusala Anupindi, Pratush Saravanan, Amalia Luthens, Amalia Bustillos, Arpita Das, Eileen de Haro, Lara Doyle-Meyers, Jayme Looper, Andrew N. Bubak, Christy S. Niemeyer, Brent Palmer, Maria A. Nagel, Vicki Traina-Dorge

**Affiliations:** 1 Department of Neurology, University of Colorado School of Medicine, Anschutz Medical Campus, Aurora, CO 80045, USA; brittany.feia@cuanschutz.edu (B.F.); kusala.anupindi@ucdenver.edu (K.A.); psaravanan@berkeley.edu (P.S.); amalia.luthens@cuanschutz.edu (A.L.); amalia.bustillos@cuanschutz.edu (A.B.); andrew.bubak@cuanschutz.edu (A.N.B.); christy.niemeyer@cuanschutz.edu (C.S.N.); maria.nagel@cuanschutz.edu (M.A.N.); 2 Cell and Developmental Biology Program, School of Medicine, Oregon Health Sciences University, Portland, OR 97201, USA; colemaco@ohsu.edu; 3 Division of Microbiology, Tulane National Primate Research Center, Tulane University, Covington, LA 70433, USA; adas@trudeauinstitute.org (A.D.); edeharo@tulane.edu (E.d.H.); ldoyle@tulane.edu (L.D.-M.); vtraina@tulane.edu (V.T.-D.); 4 Department of Veterinary Clinical Sciences, Louisiana State University, Baton Rouge, LA 70803, USA; jlooper@lsu.edu; 5 Division of Allergy and Clinical Immunology, Department of Medicine, University of Colorado Anschutz Medical Campus, Aurora, CO 80045, USA; brent.palmer@cuanschutz.edu; 6 Department of Ophthalmology, University of Colorado School of Medicine, Anschutz Medical Campus, Aurora, CO 80045, USA

**Keywords:** simian varicella virus, varicella, zoster, skin pathogenesis

## Abstract

Primary simian varicella virus (SVV) infection and reactivation in nonhuman primates is a valuable animal model in the study of varicella zoster virus disease [varicella (chickenpox) and herpes zoster (shingles)]. To understand SVV pathogenesis in skin, we inoculated 10 rhesus macaques with SVV, resulting in varicella rash. After the establishment of latency, eight of the monkeys were immunosuppressed using tacrolimus with or without irradiation and prednisone and two monkeys were not immunosuppressed. Zoster rash developed in all immunosuppressed monkeys and in one non-immunosuppressed monkey. Five monkeys had recurrent zoster. During varicella and zoster, SVV DNA in skin scrapings ranged from 50 to 10^7^ copies/100 ng of total DNA and 2–127 copies/100 ng of total DNA, respectively. Detection of SVV DNA in blood during varicella was more frequent and abundant compared to that of zoster. During varicella and zoster, SVV antigens colocalized with neurons expressing β-III tubulin in epidermis, hair follicles, and sweat glands, suggesting axonal transport of the virus. Together, we have demonstrated that both SVV DNA and antigens can be detected in skin lesions during varicella and zoster, providing the basis for further studies on SVV skin pathogenesis, including immune responses and mechanisms of peripheral spread.

## 1. Introduction

Varicella zoster virus (VZV) typically causes childhood varicella (chickenpox), establishes latent infection in multiple ganglia along the neuraxis, and reactivates decades later to produce herpes zoster (shingles), predominantly in the elderly. Varicella presents as a disseminated vesicular/macular/papular skin rash; in contrast, zoster appears as a unilateral dermatomal-distribution vesicular rash corresponding to the ganglia associated with the affected dermatomes. Due to VZV’s restricted host range, dissecting mechanisms through which VZV spreads to multiple organs, including skin, during primary infection and reactivation has been challenging. Using a SCID-hu mouse model of VZV pathogenesis, T lymphocytes have been shown to transport virus to skin from lymph nodes during primary infection [1]. However, it is unclear how VZV is transported from ganglia to multiple organs, including skin, during zoster. Earlier comparative studies of peripheral blood mononuclear cells (PBMCs) during varicella and zoster revealed that, viremia occurs at a much lower magnitude during zoster, despite abundant VZV DNA and virions detected in zoster vesicles [2,3,4,5]. Zoster skin lesions have been clinically categorized into four stages (erythematous, vesicular, pustular, and ulcerative), VZV spreads to the epidermis via sensory nerves during the erythematous stage and then to the dermis in the vesicular stage [6]. Further, virus antigens have been found mostly around the isthmus and less often near the bulb of the hair follicle [7]. Lymphocytes surrounding blood vessels have also been shown to contain VZV immediate early protein 63 (IE63), but not other structural proteins [8]. The selective detection of VZV antigens has also been reported following immunohistochemical analysis of biopsied skin samples from varicella and zoster [9]. Most studies related to VZV infection in skin have been performed on human skin biopsies because VZV is an exclusively human pathogen. Simian varicella virus (SVV) infection in non-human primates (NHP) has served as a useful model since its pathological and immunological features are similar if not identical to VZV infection in humans [10]. Upon experimental inoculation in rhesus macaques, SVV produces varicella, establishes latent infection, and can be reactivated via immunosuppression [11,12]. We previously showed that SVV antigens can be detected in sweat glands in zoster skin [13]. In this report, we extend our studies by analyzing skin scrapings and fixed skin samples (for SVV DNA and antigens, repectively), from the same rhesus macaques during both varicella and zoster.

## 2. Materials and Methods

### 2.1. Ethis Statement

Rhesus macaques that were used in this study were all housed at the Tulane National Primate Center (TNPRC) in Covington, LA. All animal housing, care, and research were performed in compliance with The Guide for the Care and Use of Laboratory Animals (National Research Council), the Animal Welfare Act guidelines, and the guidelines at the TNPRC, accredited by the Association of the Assessment and Accreditation of Laboratory Animal Care. Protocols were approved by the Institutional Animal Care and Use Committee of Tulane University (The Assurance Number A4499-01; Protocol Title: Varicella Virus Latency and Reactivation in the Nonhuman Primate, IACUC Protocol #PO177R3, 25 Oct 2021). All procedures were conducted by highly qualified veterinarians.

### 2.2. Monkeys

Ten male SVV-seronegative rhesus macaques were housed in the Tulane National Primate Center (TNPRC) in Covington, LA, and used for all experiments. The age, sex, and weight of each monkey are listed in Table 1. During acute infection and reactivation, monkeys from this study were housed in the same room, either in single or dual cages. During the immunsuppression and reactivation phase, treated monkeys, shown to be behaviorally compatable, were pair-housed with other treated monkeys. The two untreated monkeys, LT27 and LE91, were also pair-housed. A paraformaldehyde-fixed paraffin-embedded skin sample from a rhesus macaque that developed a fulminant SVV infection 53 days after irradiation (kind gift from From Dr. Steven Shipley) and an uninfected SVV-sero negative monkey (R110368) purchased from Primate biologicals (Bethesda, MD, USA) were used as positive and negative controls.

### 2.3. Acute SVV Infection and Establishment of Latency

A deltaherpesvirus strain of wild type-SVV isolated from a naturally infected monkey (*Erythrocebus patas*) was propagated in rhesus fibroblasts (Frhl-2) (ATCC, Manassas, VA, USA) and a virus stock was prepared as described previously [14]. SVV-seronegative monkeys were inoculated intratracheally with 2.4–5 × 10^5^ plaque forming units (pfu) of wild type-SVV-infected Frhl-2 cells. All monkeys were monitored following anesthesia by physical exam and blood collections every 3 to 7 days with skin scrapings and punch biopsy (4 mm) samples from skin collected upon the appearance of typical varicella rash lesions. All ten monkeys developed a typical varicella rash within 7–9 days post-inoculation (dpi) and monitoring continued until the establishment of latency confirmed by the absence of SVV DNA in PBMCs for two consecutive weeks. 

### 2.4. Immunosuppressive Regimens

**Figure 1:**
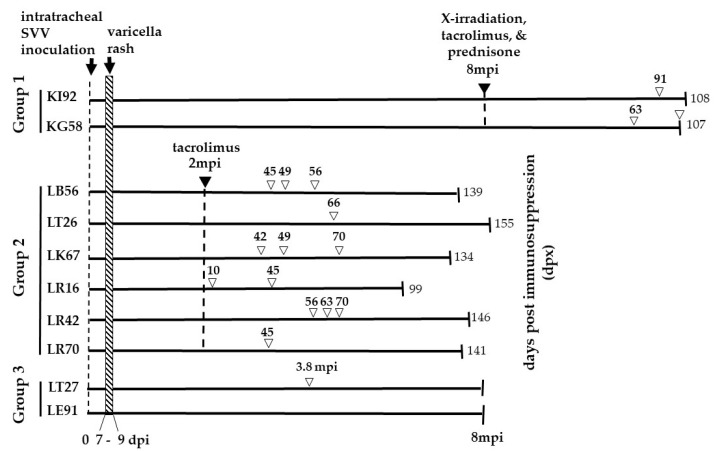
Experimental Design. The monkeys were divided into three groups. Two rhesus macaques (Group 1—KI92 and KG58) were intratracheally inoculated with 5 × 10 ^5^ pfu of SVV and developed varicella rash 7–9 days post-inoculation (dpi). Eight months’ post-inoculation (mpi), both monkeys were exposed to X-irradiation (200 cGy) and daily oral treatment with tacrolimus (80 µg/kg/day) and prednisone (1 mg/kg/day) for the duration of the experiment. KI92 and KG58 developed zoster rash, 91- and 63-days post-immunosuppression treatment (dpx), respectively. KG58 had recurrent zoster at 107 dpx. KI92 and KG58 were euthanized 108 and 107 dpx, respectively. Six rhesus macaques (group 2—LB56, LT26, LK67, LR16, LR42, and LR70) were intratracheally inoculated with 2.4 × 10 ^5^ pfu SVV, and all developed varicella rash between 7–9 dpi. Two months later, all six monkeys were treated with tacrolimus (80 µg/kg/day) daily for the duration of the experiment. All six immunosuppressed monkeys developed a zoster rash at indicated dpx (numbers above, unfilled arrowheads). Monkeys LB56, LK67, LR16, and LR42 had recurrent zoster, as indicated by multiple unfilled arrowheads. All six monkeys were euthanized at the indicated dpx. Two rhesus macaques (group 3—LT27 and LE91) were intratracheally inoculated with 2.4 × 10^5^ pfu of SVV, and both developed varicella rash by nine dpi. Neither were immunosuppressed. One of the monkeys (LT27) developed zoster rash 3.8 mpi. Group 3 monkeys were euthanized 8 mpi. DNA extracted from skin scrapings obtained at the time of zoster from monkeys in all three groups were analyzed for the presence of SVV DNA by qPCR. Euthanasia is indicated by vertical lines on the right end of the lines. The numbers at the end of the lines indicate the time (dpx (groups 1–2) or mpi (group 3)) of euthanasia.

Eight months after primary infection, establishment of SVV latency in KI92 and KG58 (group 1, Figure 1) was confirmed (absence of detectable SVV DNA in two consecutive bleeds) and immunosuppresion phase was initiated with transport of the two monkeys in a van (3-h round trip) from the TNPRC in Covington, LA, USA to the School of Veterinary Medicine, Radiation Oncology facility at Louisiana State University in Baton Rouge, LA. They were anesthetized and exposed to a single dose of 200-cGy total body X-irradiation, then treated daily with oral tacrolimus (Prograf; 500 μg; 80 μg/kg of body weight/day) and prednisone (2 mg/kg/day) until virus reactivation was confirmed, and they were euthanized 2–3 months post-zoster (Figure 1). Two months after primary infection, establishment of latency was confirmed in group 2 animals: LB56, LT26, LK67, LR16, LR42, and LR70 (Figure 1). Immunosuppresion consisted of daily treatment with oral tacrolimus (Prograf; Sandoz Inc., Princeton, NJ, USA) 80–240 μg/kg of body weight/day) until SVV reactivation was confirmed, and they were euthanized 2–3 months post-zoster. Group 3 monkeys, LT27 and LE91, were infected with SVV but not immunosuppressed or transported (Figure 1). These monkeys were also monitored for viral reactivation. All animals were monitored by physical exams every 7 days, and blood samples were collected weekly until reactivation and collected again during euthanasia.

### 2.5. Determination of Anti-SVV Antibody Levels

Anti-SVV antibody titers in serum obtained from all monkeys prior to SVV inoculation and during the time of monitoring after inoculation until euthanasia were determined using a plaque reduction neutralization test (PRNT) as described previously [12].

### 2.6. Collection and Analysis of Blood Samples for SVV DNA by qPCR

Blood samples collected from monkeys at multiple times following SVV inoculation and immunosuppression were processed using a Ficoll gradient to isolate PBMCs and a commercial DNA extraction kit per manufacturer’s instructions (Qiagen, Germantown, MD, USA), followed by quantitative PCR (qPCR) for SVV DNA in PBMCs as described previously [15].

### 2.7. DNA Extractions from Skin Scrapings and qPCR Analysis

During physical examinations, anesthetized monkeys demonstrating typical varicella lesions on the skin were identified and photographed. Single lesions were scraped using a scalpel and resuspended in 500 µL of PBS and frozen at −80 °C until DNA extraction. After thawing, cell suspensions were centrifuged at 20,000× *g* for 10 min at room temperature and the pellets were resuspended in 180 µL of ATL buffer (DNA extraction kit, Qiagen) and 20 µL proteinase-k and processed. The samples were incubated overnight at 56 °C to ensure optimal and complete lysis. DNA was extracted using Qiagen DNeasy Blood and Tissue kit according to the Quick-Start Protocol for tissues. In the final step, DNA was eluted using 70 µL of AE buffer and quantitated before qPCR using a Nanodrop spectrophotometer (ThermoFisher, Waltham, MA, USA). The DNA samples were analyzed by qPCR using primers specific for SVV open reading frame (ORF) 61 as described previously [11]. Briefly, limited dilutions of cloned SVV bacmid (containing 5000, 1000, 100, 50, 10, 5 and 1 copies of virus DNA in a background of 100 ng of salmon sperm DNA) was used in real-time qPCR using primers specific for SVV ORF61 generate a standard curve [16]. The number of copies of SVV DNA in the unknown samples were determined by comparing the C_t_ values. The C_T_ values for real-time PCR ranged from 33.8 (min) to 37.6 (max). The standard curve was linear between 1–5000 copies of SVV bacmid which was used in real-time qPCR. We performed 3 different PCR assays on each DNA extraction. A sample is considered positive only if two out of three independent PCRs are positive for SVV DNA.

### 2.8. Harvesting and Processing of Skin Samples for Immunohistochemistry

Typical papular/vesicular lesion of varicella or zoster rash in each monkey was identified. A 4-mm punch biopsy was obtained from an area different from the one used for DNA extraction. The tissues were fixed in 10% Zinc-formalin (Z-fix) (Analtech, Battle Creek, MI, USA), processed, and paraffin-embedded.

### 2.9. Immunohistochemistry

Formalin-fixed, paraffin-embedded (FFPE) skin sections (5 µm) were deparaffinized in xylene and ethanol for 15 min each. The sections were then rhydrated using graded ethanol washes and washed once with water. They were then subjected to antigen retrieval in citrate buffer (10 mM Sodium Citrate pH 6.0 and 0.05% Tween20). The citrate buffer was pre-heated in a steamer for 10 min, and the slides were submerged in the hot buffer and incubated on the benchtop for 5 min. The sections were then imunostained using the ImmPRESS kit along with Vector NovaRED substrate kit (Vector Laboratories, Burlingame, CA, USA) per the manufacturer’s instructions. The primary antibody was either rabbit polyclonal antibody raised against SVV IE63 protein (1:7000 dilution), Rabbit polyclonal antibody raised against SVV nucleocapsid (1:25,000 dilution), or Rabbit polyclonal antibody raised against SVV glycoproteins H and L (gH + L; 1:5000) (a generous gift from Dr. Wayne Gray). Normal rabbit serum (at the same dilution as the primary antibody) was used as a control. Following the first staining, some of the sections were washed with PBS for 5 min and processed using ImmPRESS Horse Anti-Rabbit IgG polymer kit, peroxidase along with Vector Blue substrate Kit, Alkaline phosphatase (Vector Laboratories) as per the manufacturer’s instructions. The primary antibody was a Mouse anti-β-III tubulin antibody (STEM CELL Technologies, Kent, WA, USA) at 1:500 dilution. Mouse IgG2a (BD Biosciences, Franklin Lakes, NJ, USA) at a dilution of 1:500 was used an isotype control. Positive controls consisted of skin sections from an acutely infected immunosuppressed rhesus macaque (B321) immunostained for SVV and β-III tubulin, which were observed under a microscope during substrate color reactions. Some of the sections were counter-stained using hematoxylin (1:10 dilution of stock) for 2 min. The slides were then mounted using glass coverslips with ProLong Gold Antifade Mountant (Life Technologies, Eugene, OR, USA) and imaged using an Olympus BX46 light mircoscope and CellSens Software (Olympus, Center Valley, PA, USA). Each staining was repeated at least three times to ensure reproducibility. 

## 3. Results

### 3.1. Primary SVV Infection in Rhesus Macaques, Establishment of Latency, and Immunosuppression

Three groups of SVV-seronegative Indian rhesus macaques were used in this study (Table 1 and Figure 1). They were inoculated intratracheally with wild-type SVV as described in the methods. All 10 monkeys developed varicella rash between 7–9 dpi and resolved thereafter. Monkeys in group 1 were inoculated with 5 × 10^5^ pfu/monkey whereas monkeys in groups 2 and 3 were inoculated with 2.4 × 10^5^ pfu/monkey. The difficulty in using consistent amounts of virus in the inoculum is due to challenges in growing SVV at high titers and batch-to-batch variation. Thus, it is possible that the differences in infection are associated with differences in the quantity of virus inoculated, but this is unlikely given that all infected monkeys developed rash. 

Earlier we demonstrated that in rhesus macaques [11,17], lack of viremia is a consistent marker for the establishment of latency. Eight month’s post infection (mpi), establishment of latency was confirmed in the two monkeys in group 1 (KI92 and KG58). They were then immunosuppressed with a single dose of X-irradiation and a combination of tacrolimus and prednisone administered daily for the duration of the experiment and were monitored for reactivation. Two months after primary infection, establishment of latency was confirmed in the six monkeys in group 2 (LB56, LT26, LK67, LR16, LR42, and LR70) and the two monkeys in group 3 (LT27 and LE91). Group 2 monkeys were treated with tacrolimus daily for the duration of the experiment. Two monkeys in group 3 (LT27 and LE91) were not treated with immunosuppressants (Figure 1). All ten monkeys were monitored for reactivation. 

### 3.2. Skin Rash during Varicella and Zoster

**Figure 2:**
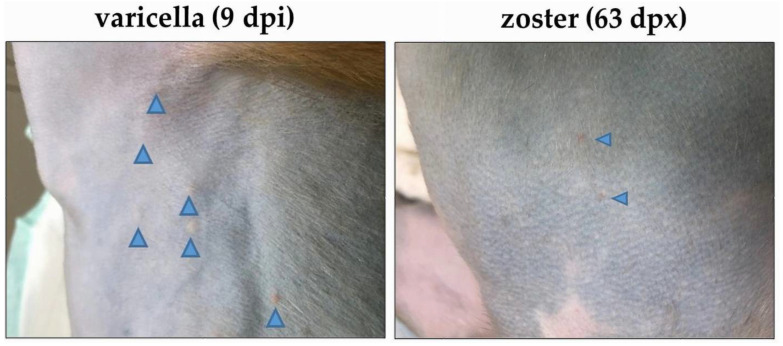
Skin rash after varicella and zoster in monkey LR42 (group 2). Varicella skin rash was seen in the torso nine days after SVV inoculation (dpi; **left image**). Zoster skin rash was seen on the torso 63 days after immunosuppression (dpx) with tacrolimus treatment (**right image**). The location of the vesicles is indicated by blue arrowheads.

The extent of varicella rash was mild in three monkeys (KI92, LR42 and LE91), moderate in two monkeys (LB56, LR16) and extensive in five monkeys (KG58, LT26, LK67, LR70, and LT27) (Table 2). At different times following immunosuppression, monkeys in all three groups developed zoster. A representative image of skin rash during varicella and zoster in LR42 (group 2) is presented in Figure 2. In all SVV-infected monkeys, the extent of rash during varicella was more pronounced with multiple lesions spread throughout the body compared to zoster, which was minimal and more localized. Unlike zoster in humans, which occurs as a dermatomal rash, both immunosuppressed and non-immunosuppressed monkeys developed zoster rash in multiple regions of the body. 

In group 1, KI92 and KG58 developed zoster 91 and 63 dpx, respectively (Figure 1). In group 2, all monkeys developed zoster between 10–66 dpx. In group 3, LT27, one of two non-immunosuppressed monkeys also developed zoster 3.8 mpi, possibly due to unrelated reduction in SVV-specific cell mediated immunity, which has been documented [13]. The other control monkey (LE91) did not develop zoster. In several of our studies, we have observed that besides immunosuppression there is a possibility that transportation, single-cage housing, and repeated removal from cages for sample collection triggered SVV reactivation [12,13,15]. One of the two monkeys in group 1 (KG58) and four (LB56, LK67, LR16 and LR42) of the six monkeys in group 2 had recurrent zoster (rash appearing one week or more after initial zoster rash had cleared). Recurrent zoster in KG58 occurred at the time of necropsy (107 dpx).

### 3.3. Detection of SVV DNA in Skin Scrapings during Varicella

DNA extracted from skin scrapings obtained during varicella in groups 2 and 3 were analyzed using real-time qPCR for the presence of SVV DNA (Table 2). During varicella, SVV DNA copy numbers in skin scrapings ranged from 50 to 1 × 10^7^ copies/100 ng. Although substantial amounts of SVV DNA were detected in most of the monkeys, the quantity of SVV DNA in skin, in general, did not always correlate with the extent of varicella rash. 

### 3.4. Detection of SVV DNA in PBMCs during Varicella and Immunosuppression

DNA extracted from sequential blood samples obtained from monkeys in all 3 groups during primary infection and later during immunosuppression, were analyzed for viremia using real-time qPCR for SVV DNA. We performed three different PCR assays on each DNA sample. Any DNA sample is considered positive only if two out of three PCRs are positive for SVV DNA During varicella, viremia peaked at 7 dpi in all monkeys, except KI92 where 26 copies of SVV DNA/100 ng) were detected at 4 dpi (Table 3). In group 1, low level viremia was detected up to 64 dpi and was not detectable at later times after the establishment of latency (data not shown). In groups 2 and 3, viremia was absent in all by 42 dpi, confirming the establishment of latency. The highest quantity of SVV DNA among all 10 monkeys was detected in both skin scraping and PBMCs of LT27 (group 3) during varicella (Table 2 and Table 3). It is possible that the detection of SVV DNA at 4 dpi could be due to input virus.

Unlike primary infection, detection of SVV DNA in PBMCs from groups 1 and 2 after treatment and immunosuppression was very sparse (Table 4). Low levels (2–10 copies/100 ng) of SVV DNA were detected three weeks after immunosuppression in five of the eight immunosuppressed monkeys (groups 1 and 2). In monkeys LK67 and LR42 (group 2), 3/12 and 3/13 sequential samples, respectively revealed low levels (3–10 copies/100 ng) of SVV DNA. 

### 3.5. SVV-Specific Antibody Response during Varicella and Zoster

Humoral response to SVV was measured using a plaque reduction (number of plaques) neutralization assay in all 3 groups of monkeys at multiple times following SVV inoculation and immunosuppression (Table 5). Anti-SVV antibody titers are expressed as the serum dilution that neutralized at least 80% of the SVV plaques compared to control cultures. In group 1, SVV-specific antibodies were detected 2 weeks post-inoculation and remained high during the establishment of latency until 70 dpi, when immunosuppression was initiated. At 14 dpx, both KI92 and KG58 showed decreases in their antibody levels that remained low even following zoster (KI92, 91 dpx and in KG58, 63 and 107 dpx; Figure 1). In group 2, all six monkeys had high antibody responses during acute infection (1:160 and 1:320) by 14 dpi that remained ≥1:80 at the start of immunosuppression. After immunosuppression, four of the monkeys (LB56, LT26, LK67, LR16) showed increased antibody levels around the time of zoster while two (LR42, LR70) remained stable (Table 5 and Figure 1). In group 3 after inoculation, LT27 had stable antibody titers (1:160–1:320) until they decreased to 1:80 at 3.8 mpi, when this monkey developed zoster and increased to 1:1280 at the time of euthanasia. In LE91, the SVV-specific antibody levels peaked at 1:80 at 14 dpi but declined, and without any zoster occurance, remained low through the course of the experiment.

### 3.6. Detection of SVV DNA in Skin Scrapings during Zoster

Typical zoster lesions were carefully observed, photographed, scraped, DNA extracted, and analyzed by qPCR for the presence of SVV DNA. The samples were analyzed in triplicate and scored as positive only if two out of three PCRs contained SVV DNA. Details about the SVV DNA-positive skin scrapings obtained during immunosuppression are presented in Table 6. Seventeen skin scrapings collected from eight monkeys during zoster contained SVV DNA. Monkey KI92 (group 1) had the highest number copies (127 copies/100 ng) in skin scrapings obtained at 91 dpx. Copies of SVV DNA in the rest of the monkeys ranged from 2–38 copies/100 ng. Skin scraping from the non-immunosuppressed monkey LT27 (group 3) with zoster contained 10 copies/100 ng. While LR42 had SVV DNA-positive skin scrapings, as well as PBMCs at 56 dpx, and LT27 was viremic at 49 dpx and had positive SVV DNA-positive skin scrapings soon after. During the reactivation phase, only 2 out of 11 blood samples that contained SVV DNA correlated with the occurrence of skin rash, suggesting subclinical reaction. 

### 3.7. Detection of SVV Antigens in Skin and Colocalization of SVV Antigens with a Neuronal Marker during Varicella and Zoster

**Figure 3:**
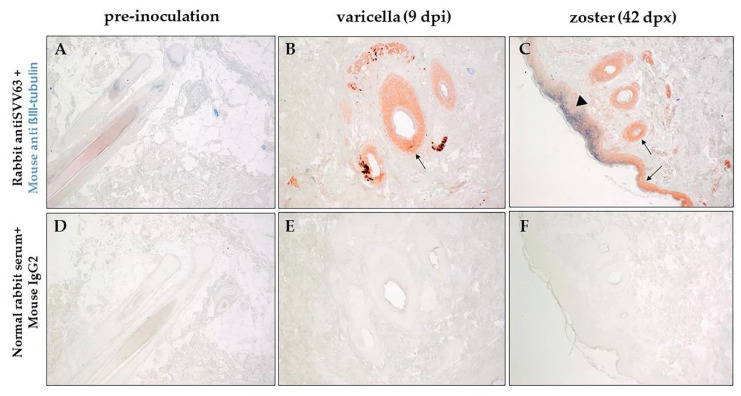
Immunohistochemical analysis of varicella and zoster skin rash from the same rhesus macaque. Sections of biopsied, zinc formalin-fixed, paraffin-embedded skin samples before inoculation with SVV (pre-inoculation), 9 dpi (days post-inoculation) and 42 dpx (days post-immunosuppression) from rhesus macaque LK67 (group 2) was analyzed using dual-color staining. Rabbit polyclonal antibodies against SVV IE63 protein (red) and mouse monoclonal antibody directed against human β-III tubulin (blue) were used as described in methods to identify the presence of virus antigen and its proximity to nerve endings. Normal rabbit serum and mouse anti IgG2a were used as negative controls (**D**–**F**). SVV IE63 protein was not detected in the pre-inoculation biopsy (**A**), but was found in hair follicles during varicella and zoster and in the epidermis during zoster (**B**, arrows). β-III tubulin was found to colocalize with SVV IE63 protein (**C**, arrowhead) in the epidermis during zoster. (Magnification, ×100).

Sections of zinc-formalin-fixed paraffin-embedded skin biopsies obtained before SVV inoculation, during varicella, during and zoster were analyzed by immunohistochemistry using rabbit polyclonal antibodies specific for SVV IE63 protein or SVV nucleocapsids and a mouse monoclonal antibody specific for βIII-tubulin (neuronal marker). Representative analysis of biopsied skin from monkey LK67 (group 2) before inoculation, nine dpi and 42 dpx are presented in Figure 3. Skin biopsy from monkey LK67 pre-inoculation did not show positive staining with rabbit anti SVV63 or mouse anti-βIII-tubulin antibodies (Figure 3A). SVV IE63 protein was detected in hair follicles (Figure 3B,C, arrows) in skin biopsies from LK67 at both nine dpi and 42 dpx. SVV IE63 protein was also detected in the epidermis and colocalized with βIII-tubulin in skin biopsy from LK67 at 42 dpx (Figure 3C, arrowhead). The concentrated βIII-tubulin colocalizing with SVV IE63 protein suggested hyperinnervation at the site of rash. Mixture of normal rabbit serum and isotype mouse IgG2a control antibodies did not show any positive staining with skin biopsies from LK67 at pre-inoculation, nine dpi, and 42 dpx (Figure 3D,E,F).

**Figure 4:**
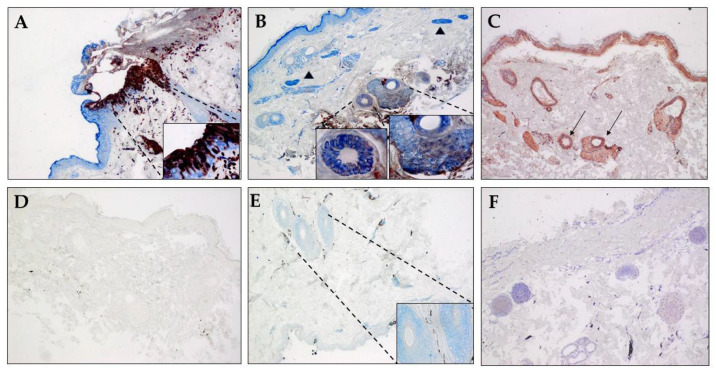
Colocalization of SVV antigen and βIII-tubulin in skin sweat glands during zoster. Sections of biopsied, zinc-formalion-fixed, paraffin-embedded skin samples from (**A**) an acutely infected immunosuppressed rhesus macaque (B321; SVV-positive control), (**B**–**D**) from rhesus macaque KI92 (group 1) 91 dpx (days post-immunosuppression), and (**E**,**F**) an uninfected SVV-seronegative rhesus macaque (R110368) were analyzed by immunohistochemistry. Rabbit polyclonal antibodies against SVV IE63 protein (red) (**A**,**B**,**E**), SVV gH + L, and (**C**) mouse monoclonal antibody directed against human β-III tubulin (blue; **A**,**B**,**E**) were used as described in methods. Nerve bundles containing βIII-tubulin are identified by arrowheads in panel B. Normal rabbit serum by itself (**F**) or with mouse anti IgG2a antibody (**D**) were used as negative controls. Colocalization of SVV IE63 protein with β-III tubulin can be seen in epidermis during acute infection (**A**, inset) and in sweat glands and hair follicles during reactivation (**B**, inset). SVV gH + L antigens can also be seen in sweat glands in an adjacent section (**C**, thin arrows). β-III tubulin, but not SVV IE63, protein can be seen in sweat glands and hair follicle in skin from an uninfected monkey (**E**, inset). Normal rabbit serum along with mouse anti IgG2a antibody on skin from zoster in monkey KI92 (**D**) or using rabbit anti SVV gH + L on skin section from the uninfected monkey (**F**) did not show any staining. Section in panel F was counterstained with hematoxylin. (Magnification, ×100, inset ×600).

A section of fixed skin obtained at necropsy from a rhesus macaque (B321) with acute SVV infection and generalized, fulminent varicella rash, after irradiation served as a positive control. The skin was analyzed using rabbit anti-SVV IE63 antibody and mouse monoclonal antibody specific for βIII-tubulin. SVV IE63 protein and βIII-tubulin were found to be colocalized in the epidermis (Figure 4A, inset), suggesting axonal spread of virus during varicella. Analysis of skin from monkey KI92 (group 1) at 91 dpx revealed colocalization of SVV and neuron-specific marker in sweat glands (Figure 4B, inset), also suggesting axonal transport of SVV to sweat glands and hair follicles during SVV reactivation. Analysis of an adjacent section of the same skin samples with rash from monkey KI92 using a mixture of rabbit polyclonal antibodies specific for SVV gH + L revealed the presence of the SVV antigens in the same sweat glands and hair follicle (Figure 4C). SVV gH + L were also seen in other areas of the skin including the epidermis. A mixture of normal rabbit serum and isotype mouse IgG2a did not show positive staining in another adjacent section of skin from monkey KI92 (Figure 4D). While SVV IE63 protein was absent in biopsied skin from the uninfected control rhesus macaque (R110368), hair follicles were positive for the neuronal marker βIII-tubulin (Figure 4E). SVV gH + L-specific antibodies did not show any positive staining in the skin section from the uninfected control monkey R110368 (Figure 4F). 

Results of immunohistochemical analyses of skin sections from all three groups of monkeys before SVV inoculation, during varicella, and zoster using antibodies specific for SVV IE63, SVV nucleocapsid, and βIII-tubulin are presented in Table 7. Overall, the SVV IE-63 protein was detected more readily than the virus proteins within the nucleocapsid. As expected, the neuronal marker (βIII-tubulin), but not SVV-specific antigens, was detected in skin sections from the uninfected control monkey (R110368). The neuronal marker was found and colocalized with SVV IE63 protein in skin in 7/8 monkeys during varicella, and 7/8 monkeys during zoster, further confirming the neuronal route of virus transport during varicella, as well as zoster. Supplementary results (Figure S1) show the presence of SVVIE63 protein in close proximity to nerve bundles and colocalizing with βIII-positive cells in epidermis in skin from monkeys during varicella as well as zoster. Some of the robust staining in the epidermis may be due to cross-reactivity with beta tubulin class II which is present in epidermal keratinocytes. 

In monkeys KI92 and KG58 (group 1), skin samples during varicella were not available, but zoster skin sections contained both SVV IE63 protein and proteins associated with SVV nucleocapsid. Detection of both SVV proteins (Table 7) and DNA (Table 6) in skin from KI92 during zoster suggested the presence of replicating virus, although skin samples for immunohistochemistry and SVV DNA qPCR were obtained from different areas of the skin. Biopsied skin during varicella rash (nine dpi) was obtained from five monkeys in group 2 (LB56, LK67, LR16, LR42, and LR70) and LT27 in group 3. SVV IE63 (an immediate-early gene) protein was detected in all six monkeys, whereas SVV nucleocapsid-specific antigens were found only in two (LR42 and LT27) of the six monkeys, likely due to higher level of expression of immediate-early genes. In two of the monkeys (LK67 (49 and 70 dpx) and LR16 (45 dpx)), biopsies were not available, but skin scrapings were found to contain SVV DNA (Table 6). During zoster, both SVV IE63 protein and proteins associated with nucleocapsids were detected in in 9/12 skin biopsies obtained from eight monkeys across all three groups. Taken together, our detection of SVV DNA in skin scrapings and SVV antigens in biopsies from all monkeys at various times, including during recurrent zoster, provided convincing evidence that SVV is associated with both varicella and zoster rash.

## 4. Discussion

**Figure 5:**
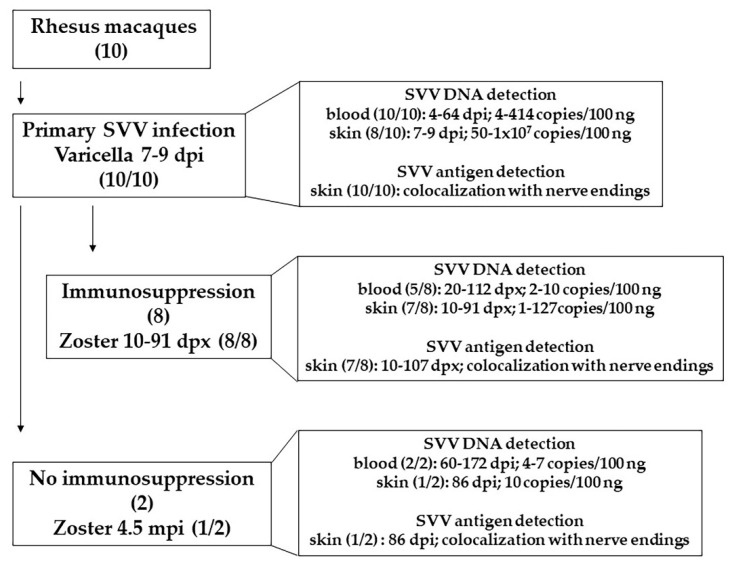
Summary of findings in this report. Ten rhesus macaques were inoculated with SVV. All of them developed varicella 7–9 dpi. SVV DNA (4–414 copies/100 ng) was detected in blood, 4–64 days post infection (dpi) in all 10 monkeys. SVV DNA (50–1 × 10^7^ copies/100 ng) was detected in skin scrapings in 8 of 10 monkeys, 7–9 dpi. Analysis of biopsied skin samples, during varicella, revealed colocalization of SVV antigens with nerve endings in all 10 monkeys. Establishment of latency was confirmed by the absence of SVV DNA in blood. Eight of latently infected monkeys were immunosuppressed. Zoster rash developed in all monkeys 10–91 days post immunosuppression (dpx). SVV DNA (2–10 copies/100 ng) was detected in blood, 20–112 dpx, in 5 of 8 monkeys. During zoster (10–91 dpx), 1–127 copies of SVV DNA (per 100 ng) were detected in skin scrapings. In the affected skin, SVV antigens were found to be colocalized with nerve endings. In two monkeys were not immunosuppressed, SVV DNA (4–7 copies/100 ng) was detected 60–172 dpi. One of the two non-immunosuppressed monkey developed zoster, 4.5 mpi (months post infection). SVV DNA (10 copies/100 ng) and antigens was detected in skin scrapings during zoster (86 dpi). SVV antigen was found to be colocalized with nerve endings. Abundance of SVV DNA and antigens in skin was substantially higher during varicella compared to zoster. SVV infection and reactivation in rhesus macaques serves as an extremely useful model to study varicella and zoster in humans.

Like VZV infection in humans, SVV causes varicella becomes latent, and reactivates to produce zoster in NHPs. Key findings in the report are summarized in Figure 5. Viremia and the extent of rash varied among the 10 monkeys (Table 2), probably due to the differences in their antiviral response. Peak viremia and varicella rash occurred at the same time (7–9 dpi) in all monkeys except one (KI92), in which peak viremia occurred at four dpi (Table 3). Extensive rash was seen during varicella (Figure 2), due to the hematogenous virus spread as indicated by high viremia (Table 3) [18]. There was not a complete correlation between the severity of varicella rash and SVV DNA in skin scrapings (Table 2). However, this observation was likley due to differences in sampling as scrapings and biopsies were obtained from from distinct single lesions, mostly from different areas of the body. Once establishment of latency is confirmed by the absence of detectable virus DNA in blood for two consecutive weeks, immunosuppression was initated. A limitation of this study is that animals were immunosuppressed with a combination of tacrolimus, irradiation, and steroids to trigger reactivation. In humans, reactivation occurs with a decline in cell mediated immunity (CMI) associated with aging and in the setting of immunosuppression such as seen in cancer and transplant patients treated with similar therapies (tacrolimus, irradiation, steroids). Because we could not indefinitely age the animals and wait for reactivation, our model immune suppresses to decrease VZV-specific CMI. 

Monkeys in groups 1 and 2 were immunosuppressed at eight and two mpi, respectively, after viremia was absent for two weeks. Viremia following immunosuppression and during zoster was intermittent and much lower than viremia during primary infection, probably due to reduced hematogenous spread of virus and increased axonal spread following reactivation from individual ganglia (Table 4). Skin rash during zoster was also much less extensive compared to varicella (Figure 2). Lack of detectable viremia during zoster compared to varicella despite the presence of virus DNA in skin rash vesicles in humans have been documented [2]. As observed before, no significant changes in SVV-specific antibodies were observed in immunosuppressed monkeys during zoster, suggesting that humoral response is unlikely to play an important role in virus reactivation [12,13,15,19]. However, in one monkey (LT27, group 3) that was not immunosuppressed, we noticed a reduction in the virus-specific antibodies at the time of zoster, the reason for which remains unclear (Table 5). In the other non-immunosuppressed monkey (LE91-group 3), the antibody response after inoculation was low likely due to the relatively low-level viremia seen at seven dpi (Table 3). 

Our detection of SVV DNA in skin scrapings from all eight immunosuppressed monkeys at various times provided convincing evidence that presence of SVV corrlates with zoster rash. Recurrent zoster seen in seven out of eight immunosuppressed monkeys from groups 1 and 2 confirmed our earlier observation and what has been found in humans during VZV reactivation [13,20,21]. Monkey KI92 (group 1) had substantially higher copies of SVV DNA in skin scraping at 91 dpx (127 copies/100 ng) possibly due to the 

Multiple modes of immunosuppression used, compared to only tacrolimus-treated monkeys in group 2 (Table 6). Detection of higher quantities of SVV DNA in zoster skin (91 dpx in KI92 and 70 dpx in LR42; Table 6) in the absence of viremia at these times (Table 4) further supports the notion of axonal spread of SVV during reactivation. Earlier, we documented that during varicella, SVV enters the ganglia before the appearance of skin rash [22]. Colocalization of SVV antigens with the neuronal marker in skin, along with high level of viremia, suggests either simultaneous hematogenous spread and axonal transport of virus to skin or spread of infection between keratinocytes during varicella. On the other hand, detection of minimal viremia, as well as SVV DNA in skin rash combined with colocalization of SVV antigen with the neuronal marker, supports the idea of virus being transported preferentially via the axonal route from the ganglia to the skin during reactivation and zoster. 

While we detected SVV IE63 protein in all the varicella and zoster skin biopsies, proteins associated with SVV nucleocapsids were not detectable in two varicella and three zoster skin biopsies. Detection of some, but not other, VZV proteins in skin biopsies from varicella and zoster in humans has been reported [9]. Selective detection of VZV IE63 protein compared to glycoproteins in early zoster lesions has also been attributed to the reduced abundance of glycoproteins and preferential resistance of IE63 protein to proteolysis [23]. During zoster in humans, VZV gC has been found in axons and Schwann cells in skin biopsies [9], and VZV IE63 protein and glycoproteins have been seen in dermal nerves and perineurial dendrocytes [23]. SVV antigens were found to be colocalized with the neuronal marker in most, if not all, of the skin samples analyzed. Our detection of SVV antigens associated primarily with sweat glands and hair follicles was very similar to VZV antigens in human skin during zoster [6,7,8,23,24,25]. Together, our results show that compared to primary SVV infection (varicella), SVV causes a milder skin rash and less viremia during zoster. In addition, SVV DNA and antigens can be detected in both skin scrapings and biopsies. Huch et al. [26] showed that during zoster in humans, Langerhans and plasmacytoid dendritic cells are strongly associated with VZV antigen-positive cells in skin. The lack of availability of rhesus specific reagents have hampered the progress in this area. Our future studies will include identification of antibodies that cross react with NHP skin to understand the role of immune cells in controlling zoster rash, as well as neuronal subtypes that are involved in pathological manifestations, such as postherpetic neuralgia.

## Figures and Tables

**Table 1:** List of monkeys used in this study.

Group	Monkey ID	Age ^a^	Sex	Weight (Kg)	Primary Infection (dpi ^b^)	Reactivation	
						Treatment	Dpx ^c^/mpi ^d^
1	KI92	4.0	M	9.9	7	A ^e^	84
	KG58	4.2	M	9.5	9	A	63
2	LB56	6.7	M	8.2	9	B ^f^	45
	LT26	4.3	M	4.3	9	B	66
	LK67	4.7	M	8.1	9	B	42
	LR16	4.4	M	8.3	9	B	10
	LR42	4.4	M	8.0	9	B	56
	LR70	4.4	M	9.2	9	B	45
3	LT27	4.3	M	8.3	9	C ^g^	3.8 ^d^
	LE91	6.6	M	10.5	7	C	-
control	B321	3.0	M	5.9	D ^h^	E ^i^	56
control	R110368	5.0	M	U ^j^	NA ^k^	NA	NA

^a^ Years. ^b^ Days post-inoculation. ^c^ Days post-immunosuppression. ^d^ months post inoculation, ^e^ A-Irradiation, tacrolimus, prednisone. ^f^ B-Tacrolimus. ^g^ C-Not immunosuppressed, ^h^ D-time of natural primary infection unknown, ^i^ E-Irradiation, ^j^ Unknown, ^k^ Not applicable.

**Table 2:** Severity of varicella (7–9 dpi) rash and detection of SVV DNA in skin scrapings.

Group	Monkey ID	Severity ^a^	SVV ORF 61 DNA (Copy #/100 ng)
1	KI92	2+	NA ^b^
	KG58	4+	NA
2	LB56	3+	50
	LT26	4+	5 × 10^5^
	LK67	4+	1 × 10^6^
	LR16	3+	310
	LR42	2+	6 × 10^4^
	LR70	4+	2 × 10^3^
3	LT27	4+	1 × 10^7^
	LE91	2+	4 × 10^5^

^a^ The type and severity of the rash is graded as macular, papular, vesicular, and hemorrhagic. Scoring parameters are based on number of lesions with 2+ = 6–10; 3+ = 11–20; 4+ = >21. ^b^ Not available.

**Table 3:** Viremia during varicella.

Group	Monkey ID	SVV ORF 61 DNA Copy #/100 ng ^a^								
		Days Post-Inoculation								
		4	7	9/10	11	14	21	29/30	42	64
1	KI92	26	4	6	20	0	0	0	NA ^b^	1
	KG58	78	161	20	12	2	1	0	NA	3
2	LB56	0	95	0	NA	0	0	0	0	0
	LT26	21	103	9	NA	0	0	0	0	0
	LK67	0	103	31	NA	9	17	6	0	0
	LR16	7	36	8	NA	0	0	0	0	0
	LR42	7	282	40	NA	7	3	0	0	0
	LR70	21	128	16	NA	0	0	0	0	0
3	LT27	0	414	47	NA	0	0	0	0	0
	LE91	0	23	0	NA	0	0	0	0	0

^a^ All samples done in triplicates and averaged. ^b^ Not available.

**Table 4:** Viremia during SVV reactivation.

Group	Monkey ID	SVV ORF 61 DNA Copy #/100 ng ^a^												
		Days Post-Immunosuppression Treatment (dpx)												
		Pre	5/7	12/14	19/21	28	35	42	49	56	63	70	84	112
1	KI92	0	0	0	2	0	3	0	0	0	0	0	0	NA ^b^
	KG58	0	0	0	0	0	0	0	0	0	0	0	0	NA
2	LB56	0	0	0	0	0	0	0	0	0	NA	0	0	0
	LT26	0	0	0	0	0	0	0	0	0	0	0	0	0
	LK67	0	0	0	8	3	0	0	6	0	NA	0	0	0
	LR16	0	0	0	3	0	0	0	0	0	NA	0	0	NA
	LR42	0	0	0	10	0	0	0	0	5	0	0	0	3
	LR70	0	0	0	0	0	0	0	0	0	NA	0	2	3
		Months post infection (mpi)												
		2	2.2	2.4	2.6	3	3.2	3.4	3.6	3.8	4.1	4.3	4.8	5.7
3	LT27 ^c^	0	0	0	0	0	0	0	7	0	0	0	4	0
	LE91 ^c^	0	0	0	6	0	0	0	0	0	0	0	0	6

^a^ All samples done in triplicates and averaged. ^b^ Not available. ^c^ Not immunosuppressed.

**Table 5:** Neutralizing antibody titers during varicella and zoster.

Group	Monkey ID	Anti-SVV Antibody Titer ^a^								
		PRE	Dpi ^b^			Dpx ^c^				Necropsy
			14	28	70	14	42	56	70	
1	KI92	<1:10	1:160	1:160	1:320	1:40	1:80	NA ^d^	1:40	1:40
	KG58	<1:10	1:160	1:160	1:320	1:160	1:160	NA	1:40	1:80
2	LB56	<1:10	1:160	1:160	1:160	1:160	1:80	1:320	1:160	1:320
	LT26	<1:10	1:160	1:160	1:640	1:320	1:320	1:640	1:320	1:640
	LK67	<1:10	1:160	1:80	1:80	1:80	1:160	1:160	1:80	1:160
	LR16	<1:10	1:160	1:80	1:160	1:80	1:80	1:160	1:80	1:160
	LR42	<1:10	1:320	1:320	1:160	1:320	1:320	1:320	1:160	1:320
	LR70	<1:10	1:160	1:80	1:160	1:160	1:160	1:160	1:160	1:320
		Months post inoculation (mpi)								
		PRE	0.5	1.0	2.3	2.5	3.4	3.8	4.3	8.0
3	LT27 ^e^	<1:10	1:320	1:320	1:160	1:320	1:160	1:80 ^f^	1:80	1:1280
	LE91 ^e^	<1:10	1:80	1:40	1:20	1:40	1:20	1:40	1:20	1:40

^a^ Anti-SVV antibody titers are expressed as the serum dilution that neutralized at least 80% of the SVV plaques. compared to control cultures. ^b^ Days post-inoculation. ^c^ Days post-immunosuppression. ^d^ Not available. ^e^ Not immunosuppressed. ^f^ Time of zoster.

**Table 6:** Detection of SVV DNA in skin scrapings during zoster.

Group	Monkey ID	SVV ORF 61 DNA Copy #/100 ng ^a^									
		Days Post-Reactivation (dpx)									
		10	42	45	49	56 ^b^	63	66 ^b^	70	91	107
1	KI92									127	
2	LB56			1	6	16, 9					
	LT26							1, 6			
	LK67		2		3				5		
	LR16	6		4							
	LR42					2	12		38		
	LR70			3							
		Months post inoculation (mpi)									
						3.8					
3	LT27 ^c^					10					

^a^ All samples done in triplicates and averaged. ^b^ Samples from two locations were analyzed. ^c^ Not immunosuppressed.

**Table 7:** Detection of SVV DNA and antigens in skin of rhesus macaques during varicella and zoster.

	Monkey ID	Uninfected	Pre- infection	Acute Infection (dpi)	Reactivation (dpx/mpi ^a^)	Antiserum		
Group						SVV IE 63	SVV Nucleo-Capsid	ß-III Tubulin
	R110368	SVV- ^b^		NR ^c^	NR	-	-	+
1	KI92			NA ^d^	91	+	+	+
	KG58			NA	107	+	+	+
2	LB56		SVV-			- ^e^	-	+
				9		+	-	+
					45	+	+	+
					49	+	-	+
					56	+	-	+
	LK67		SVV-			-	-	+
				9		+	-	+
					42	+	+	+
					49	NA	NA	NA
					70	NA	NA	NA
	LR16		SVV-			-	-	+
				9		+	-	+
					10	+	+	+
					45	NA	NA	NA
	LR42		SVV-			-	-	+
				9		+	+	+
					56	+	-	+
					63	+	+	+
					70	+	+	+
	LR70		SVV-			-	-	+
				9		+	-	+
					45	+	+	+
3	LT27		SVV-			-	-	+
				9		+	+	-
					3.8 ^a^	+	+	+

^a^ Months post primary infection. ^b^ SVV- denotes sero-negative animal. ^c^ Not relevant. ^d^ Not available. ^e^ Background staining seen. +, denotes a positive antibody reaction.

## Data Availability

Not applicable.

## Supplementary Materials

The following supporting information can be downloaded at: https://www.mdpi.com/article/10.3390/v14061167/s1, Figure S1: Detection of SVV IE63 protein and βIII-tubulin in epidermis and nerve bundles in skin during varicella and zoster in rhesus macaques.
