# Supplementary material for: Simian Varicella Virus Pathogenesis in Skin during Varicella and Zoster
Source: Viruses. 2022 May 27;14(6):1167. doi: 10.3390/v14061167 (PMC9227806; doi:10.3390/v14061167)
Supplement: Supplementary file 1 [file viruses-14-01167-s001.zip › viruses-1683087-supplementary.pdf]

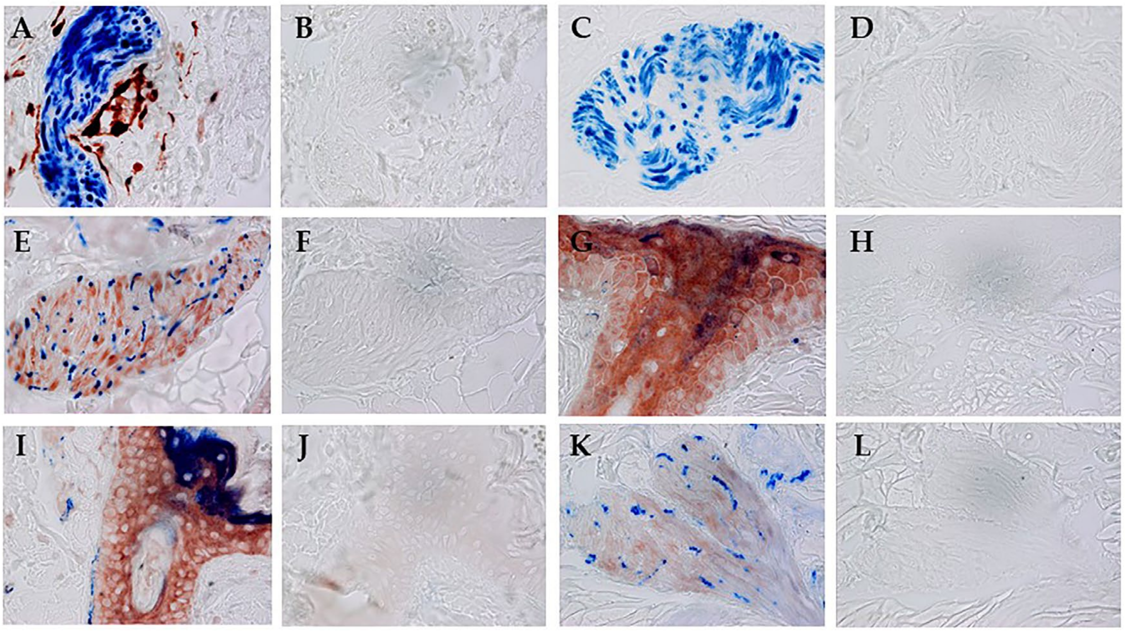

Supplementary Figure S1. Detection of SVV IE63 protein and βIII-tubulin in epidermis and nerve bundles in skin during varicella and zoster in rhesus macaques. Skin sections from acutely infected monkey B321(**A, B**), uninfected monkey R110368 (**C, D**), LR70, 9 dpi (**E, F**), 45 dpx (**G, H**), LB56, 9dpi (**I**, **J**) and 49 dpx (**K**, **L**) were analyzed by immunohistochemistry using either polyclonal antibody raised against SVV IE63 protein and mouse anti βIII-Tubulin antibody (**A, C, E, G, I**, **K)** or normal rabbit serum and mouse anti isotype IgG2a antibody (**B, D, F, H, J, L**) as described in methods. SVV IE63 protein (Brown) can be seen in close in close proximity to nerve bundles (blue) in skin sections from acutely infected monkey B321(**A**), in skin during varicella in LR70 (**I**) and during zoster in LB 56 (**K**). βIII-tubulin containing nerve bundles but not SVV IE63 protein were seen in skin from the uninfected monkey R110368 (**C**). SVVIE63 protein was also found to be colocalized in epidermal cells in skin during varicella in LB56 (**I**) and during zoster in LR70 (**G**). Normal rabbit serum along with mouse anti IgG2a antibody did not show positive staining in any of the skin sections (**B, D, F, H, J, L**) (Magnification , ×60).
